# Modifiers of Hearing Impairment in Humans and Mice

**DOI:** 10.2174/138920210791233054

**Published:** 2010-06

**Authors:** Denise Yan, Xue-Zhong Liu

**Affiliations:** Department of Otolaryngology, Miller School of Medicine, University of Miami, Miami, FL 33136, USA

**Keywords:** Deafness, heredity, phenotypic variation, heterogeneity, allelism, modifier genes, digenic inheritance, inbred mouse strain.

## Abstract

Lack of penetrance and variability of expression are common findings in nonsyndromic hearing loss with autosomal dominant mode of inheritance, but are also seen with recessive inheritance. Now we know that genotype cannot necessarily predict phenotype due to the complexity of the genome, the proteome interacting with the transcriptome, and the dynamically coupled systems that are involved. The contribution of genetic background to phenotypic diversity reflects the additive and interactive (epistasis) effects of multiple genes. Because, individual genes do not act alone but rather in concert with many other genes, it is not surprising that, modifier genes are common source of phenotypic variation in human populations. They can affect the phenotypic outcome of a given genotype by interacting in the same or in a parallel biological pathway as the disease gene. These modifier genes modulate penetrance, dominance, pleiotropy or expressivity in individuals with Mendelian traits and can also be exerted by influencing the severity, the penetrance, the age of onset and the progression of a disease. In this review, we focus on modifier genes that specifically affect hearing loss phenotypes in humans as well as those described in mice. We also include examples of digenic inheritance of deafness, because additive or interactive effects can also result from interaction between two mutant genes.

## INTRODUCTION

Phenotypic variation in heritable forms of hearing impairment has been reported within families [1-4] and in individual probands carrying the same geneticmutations [5]. The heterogeneity of genes causing hearing loss is only one factor contributing to phenotypic heterogeneity. Mutations in one hearing loss- associated gene may also lead to multiple phenotypes, which are known as allelism. In addition, phenotypic variation may result from physiological interaction of environmental factors and / or stochastic developmental events, as well as from genetic modifier.

Hearing loss (HL) is the most common sensory deficit in humans, affecting 1 in 1,000 newborns and over 50 percent of individuals 80 years of age or older. The World Health Organization (WHO) has estimated in 2005 that, 278 million people worldwide have moderate to profound hearing loss in both ears (WHO; http://www.who.int/mediacentre/fact-sheets/fs300/en/index.html). Results of the 2002 National Health Interview Survey estimate that nearly 31 million of all noninstitutionalized adults (aged 18 and over) in the United States have hearing disability [6]. Approximately 50% of the cases of congenital hearing loss are thought to be attributable to genetic causes and the remainder to environmental factors. In a particular population, the relative contribution of the environmental causes may be determined by social factors, such as population structure and rates of consanguineous marriages, infection control and neonatal and postnatal medical care provision [7]. Thus, in countries with poor health care, environmental causes of deafness may outnumber those attributed to genetics, whereas in developed countries, the proportion of cases with a clear environmental etiology is likely to decline as better therapies for bacterial and viral infections are provided, acoustic trauma in the workplace is recognized and prevented, and ototoxic drugs are avoided. Though, a major health concern costing more than $30 billion annually in lost productivity, special education, and medical treatment [8], hearing loss is often minimized or ignored.

HL is based on ascertainment of three attributes: type of hearing loss (the site of pathology in the auditory pathway), degree of hearing loss (the extent to which hearing is impaired), and configuration of the hearing loss (the frequencies affected). There are three basic types of HL: conductive hearing loss is usually associated with outer or middle ear pathology, while sensorineural hearing loss occurs when there is damage to the inner ear (cochlea) or to the nerve pathways from the inner ear (retrocochlear) to the brain. Mixed hearing loss is a combination of conductive and sensorineural HL. Based on age at onset of deafness, HL can be described as prelingual or postlingual. Approximately, two-third of prelingual cases have a ***genetic*** basis [9]. The remaining one-third of cases can be attributed to environmental and unidentified genetic factors. In most cases, inherited HL is monogenic. In 70% of cases, HL occurs as sole defect and is classified as nonsyndromic. In the remaining 30%, the HL is accompanied by other medical or physical findings and is called syndromic [10]. There are several ways in which monogenic hearing loss can be inherited. Autosomal recessive non-syndromic hearing loss (ARNSHL) is the most prevalent accounting for 80% of the cases and is typically prelingual, while non-syndromic autosomal dominant HL (ADNSHL) accounts for about 20% of cases and is most often postlingual. In 2-3 % of cases, the inheritance is either X-linked or mitochondrial [11].

The genetic basis of hearing loss is complex. Mutations in some genes can lead to both recessive and dominant forms of deafness; alternatively, different mutations in the same gene may result in syndromic and nonsyndromic deafness [12]; and even within a single gene, the same mutation can give rise to quite variable phenotypes [13]. In some recessive hearing loss cases, the combination of two mutations in different genes from the same functional group may be the cause of the hearing loss [13-15]. This phenotypic heterogeneity demonstrates how the type of mutation within the gene and its allelic combinations can affect the overall clinical presentation, complicating therefore the establishment of genotype-phenotype correlations. Moreover, recent observations indicate that the phenotypic heterogeneity observed for a given mutation may also be due to the contribution of modifier genes.

Different genetic sources can account for the wide variety of phenotypes observed in individuals with hereditary hearing loss, including genetic heterogeneity, allelic heterogeneity, and genetic background effects or modifier loci. In humans, distinguishing among these sources of variability can be difficult. However, because of the similarity between the human and other mammals’ genomes and due to the conservation of the genes during mammalian evolution, the identity and location of candidate modifier genes in human can be reliably predicted from studies in model organisms. Here, we review the evidence for modifier genes that specifically affect hearing disorders in both humans and mice. Because, difference between modifier genes and diginic inheritance is not always clear, we have included in this review, several examples of digenic inheritance of deafness that have been reported in both humans and mice. 

### Ear and Sound Processing

The mammalian inner ear is a highly intricate organ and is composed of two sensory systems: the cochlea embedded in perilymph, processes auditory signals and the vestibular apparatus, consisting of the vestibule and semicircular canals, is responsible for postural equilibrium. While, cochlear defects cause hearing loss, vestibular dysfunction, is manifested as vertigo or dizziness in humans and circling behavior in mice [16] and zebrafish [17]. Different cell types can be distinguished in the sensory epithelia of the cochlea or the Organ of Corti and in the vestibule: sensory cells, known as hair cells; supporting cells and neurons. There are two types of hair cells; a row of inner hair cells and three rows of outer hair cells. The inner hair cells are pure receptor cells that are primarily responsible for transmission of signals to the acoustic nerve and the auditory cortex. The outer hair cells have both sensory and motor capabilities that contribute to hearing sensitivity and frequency selectivity by amplifying sound reception [18]. Each hair cell has in its apical pole a bundle of stereocilia, packed with actin filaments and linked to each other at their tips. Stereocilia are the mechanoreceptive structure of the hair cells as they deflect in response to sound vibration. The stereocilia are bathed by endolymph, whereas the basolateral cell surfaces of the hair cells are bathed by perilymph. The tectorial membrane is a sheet of extracellular matrix that overlies the sensory epithelium of the cochlea. Deflection of a stereocilia bundle in response to sound waves opens mechanically-gated ion channels, resulting in an influx of potassium (K^+^) from the potassium-rich endolymph into the sensory cells. The influx of K^+^ ions causes a change in membrane potential proportional to the intensity of the acoustic stimulus. This depolarization of hair cells then activates calcium channels, leading to calcium influx into the hair cells. This calcium influx triggers the release of neurotransmitters into postsynaptic terminals that in turn activate the acoustic nerve. The hair cells are repolarized when the K^+ ^ions leave these cells *via *potassium channels and are taken up by the epithelial supporting cells. The K^+^ ions are then passively diffused to the stria vascularis through gap junctions and are actively pumped back into the endolymph through potassium channels, thereby resetting the mechanoelectrical transduction system [19-22]. Defect in either of these auditory processing components leads to hearing impairment.

### Genetic Heterogeneity - A Hallmark of Hereditary Deafness 

Hearing is a complex process, thus, it should come as no surprise that the causes of hearing loss are also complex and that hereditary hearing disorders exhibit a high degree of genetic heterogeneity. It is estimated that at least 1% of human protein-coding genes (i. e. about 300 genes) are involved in perception of sound [23], and they are thus potential candidates to cause hearing loss when mutated. There are over 400 distinct syndromes with deafness as one of the components have been described (www.ncbi.nlm.nih.gov/omim) and the underlying genetic defect for many of the most common forms have been identified. However, the vast majority (>70%) of inherited hearing disorders are non-syndromic [7].

Despite the fact that the genetic basis of hearing loss has been studied for decades, identification of the causative genes began only in the 1990s and molecular mechanisms underlying deafness have just started to be unraveled. The main obstacles to successful finding of genetic defects leading to hearing loss included: (i) inaccessibility of the cochlea, being buried within the temporal bone, (ii) extreme genetic heterogeneity, (iii) absence of distinctive clinical features for the various gene defects, and (iv) assortative mating. Nevertheless, the past few years have witnessed remarkable achievements in identifying genes associated with deafness. The identification of these genes and functional characterization of the proteins they encode has provided a major insight into our knowledge of the physiology and pathophysiology of the auditory system. There are approximately 113 mapped loci and 51 different genes that have been shown to underlie hereditary sensorineural nonsyndromic hearing loss (NSHL) in humans (http:webhost.ua.ac.be/hhh/). Among the different loci reported, 46 are autosomal dominant, 53 for autosomal recessive and 5 for X-linked deafness. In addition, a Y chromosome-linked locus and two genes on the mitochondrial genome have to be added to the list of genes responsible for NSHL [24,25]. There are mouse models for thirty-five of the 51 deafness causing genes, and for half of them, the link to hearing loss was identified prior to or concurrently with their identification in humans [26]. There are in addition, over 130 protein-coding genes that have not been implicated in deafness in humans thus far have been identified as responsible for hearing loss in mice. Mouse models of genetic HL have enabled researchers to characterize the cellular and molecular deficits involved in many of these genetic mutations, including a few models with double mutations (i.e., mutations in two different genes interact to cause hearing loss, while each mutation alone does not). Perhaps more importantly, they have provided insight into the normal and impaired auditory system.

## MODIFIERS OF HEARING LOSS IN HUMANS

Genetic modifiers are one of the several factors that can contribute to the phenotypic diversity of a genetic disorder and complicate the genetics of mendelian traits. Products of modifier genes may include proteins that physically interact with the mutant protein, factors that influence the timing or rate of transcription of the mutated gene, factors that affect the stability of mutant protein. Modifiers decrease the effects attributable to the target gene that is the mendelian variant and enhance those attributable to the modifier gene. 

### Modifying Factors for the Phenotype Linked to Mitochondrial DNA ( mtDNA ) Mutations

Many studies showed that the generation of reactive oxygen species (ROS) and free radicals are involved in the cascade of cochlear events culminating in acoustic trauma [27]. Intracellular ROS are primarily generated by the mitochondrial electron transport chain, making the mitochondria a prime target of oxidative damage. However, the high mutation rate in the mtDNA is not only due to the oxidative damage, but also caused by the lack of protective action by histones and the limited capacity of an efficient DNA repair system [28,29]. The mutation rate of mtDNA has been estimated to be as much as two orders of magnitude greater than that for the nuclear DNA [30]. In the case of mtDNA mutations both mutated as well as normal mitochondrial may be present within the same cell (heteroplasmy) or a particular mitochondrial mutation may be present in all mitochondrial DNA molecules of an organism (homoplasmy). 

Various mtDNA mutations causing progressive nonsyndromic hearing loss have been identified. Mutations in the 12S ribosomal RNA (rRNA) and tRNASer(UCN) genes account for the most cases of maternally inherited nonsyndromic deafness [31]. Nonsyndromic deafness-associated mtDNA mutations occurs often in homoplasmy state or at high levels of heteroplasmy, suggesting a high threshold for pathogenicity [32]. The human mitochondrial 12S rRNA A1555G mutation is the most common and has been associated with and without aminoglycoside-induced and nonsyndromic deafness in many families of different ethnic backgrounds [33-39]. It has been detected in 0.6-2.5% of the Caucasian population with nonsyndromic hearing loss, and the frequency is found particularly high in the Spanish population [34]. In the Asian populations, the prevalence of A1555G mutation is also high: 2.9% in Chinese [35], 3% in Japanese [40] and 5.3% in Indonesia [38].

Phenotypic manifestation of mutation A1555G is extremely variable, including cases of normal hearing carriers [41]. Hearing loss, in affected individuals, varies in several respects, including age at onset and severity; for some individuals, hearing loss is present at birth, whereas others exhibit a slow, progressive hearing loss of adult-onset. Aminoglycoside antibiotics, mitochondrial haplotypes and nuclear modifying genes have been proposed as the three major modulators for the phenotypic expression of the deafness-associated 12S rRNA mutations [31].

In cases of no previous history of exposure to aminoglycosides, the A1555G mutation induces a clinical phenotype that varies substantially among family members and ranges from severe congenital deafness, to moderate progressive hearing loss of later onset, to completely normal hearing [31]. The penetrance has also been shown different between families carrying the A1555G mutation. In some families, most of the individuals carrying the A1555G develop hearing loss, but in others, the penetrance can be extremely low [39,42]. Functional characterization demonstrate that more severe biochemical defects were observed in the mutant lymphoblastoid cell lines derived from symptomatic individuals than from cell lines derived from asymptomatic individuals in a same family [43]. However, under a constant nuclear background, a nearly identical degree of mitochondrial dysfunction was observed in cybrid cell lines derived from symptomatic and asymptomatic individuals [44]. These findings strongly indicate that the A1555G mutation is a primary cause of deafness and that nuclear modifier genes play a role in modulating the phenotypic expression of the hearing loss associated with the A1555G mutation. 

Several mitochondrial genes (tRNASer(UCN), tRNAGlu, tRNAArg, tRNAThr) and a few nuclear genes have subsequently been reported as being genetic modifiers [45-48]. Members of Arab-Israeli, Spanish and Italian families carrying the A1555G and homozygous for A10S TRMU mutation have been shown to exhibit prelingual profound deafness [45]. Functional analysis indicated that the homozygous A10S mutation in the *TRMU* gene disrupts the mitochondrial tRNA metabolism, specifically lowering the steady-state levels of mitochondrial tRNA and thereby contribute to the impairment of mitochondrial-protein synthesis [45,46]. Mutations in the *GJB2* gene have also been implicated in modulating the severity of hearing loss associated with the A1555G mutation. The modulating effect might be explained by the inability of cochlear cells to efficiently utilize metabolites for energy production which in turn may affect the normal turnover rates of gap junction proteins [49]. A possible nuclear modifier gene associated with hearing loss without exposure to aminoglycosides in subjects with the A1555G mutation has been localized to chromosome 8p23.1 [50], but the identification of this gene has remained elusive. The mitochondrial transcription factor B1 (*TFB1M*), as well as the human homologs of yeast MTO1 and MSS1 genes, *MTO1* and *GTPBP3*, respectively, have also been reported to be involved in the process of mitochondrial RNA modification [51,52]. However the precise mutation (s) and /or variant (s) responsible for the modifier effect of these genes remain to be determined.

### *GJB2* (*CX26*) Gene Mutations Show Phenotypic Variation

GJB2, which encodes the gap junction protein connexin 26 (Cx26) is responsible for up to 50% of the cases with autosomal recessive non-syndromic sensorineural hearing loss (DFNB1) [53]. Cx26 is expressed in the spiral ligament, the basal cells of the stria vascularis, various supporting cells, and the limbal fibrocytes [54,55]. Cx26 is presumed to mediate the recycling of potassium ions that flow into sensory hair cells as part of the transduction current.

There are over 100 different mutations of *GJB2* that cause non-syndromic deafness and a significant difference in the frequency and distribution of the mutations have been observed in different populations (http://davinci.crg.es/deafness/index.php). Most interestingly, a single mutation, 35delG, accounts for up to 70% of the Northern and Southern European, as well as American Caucasian populations with *GJB2 *mutations, with a carrier frequency ranging from 1.3 to 2.8% [56,57]. In the Jewish deaf population, the 167delT, accounts for 40% of the pathologic alleles [58] and has 4% carrier frequency among Ashkenazi Jews [59]. The 235delC mutation is the most prevalent in the Eastern Asian (Japanese, Chinese and Korean) populations. The R143W mutation has been associated with recessive nonsyndromic deafness in Ghana and the W24X, the most common *GJB2 *mutation in India, has also been found in high prevalence in Slovak and Spanish gypsies (www.gendeaf.org). A series of reports have attempted to assess a possible genotype-phenotype correlations for *GJB2 *mutations [5,11,60-62]. A multicentre study by Snoeckx and colleagues [63] establishes some genotype- phenotype relationships, including the observation that probands with biallelic frameshift or nonsense mutations had more severe hearing impairment than those with biallelic missense mutations. In addition, the M34T and V37I variants have shown to induce a mild hearing loss when in compound heterozygosity with a truncated mutation. Although, the phenotype expression can be predicted on the basis of the genotype for most genotypes, the correlation could only partly explain the phenotypic variability. For the majority of these genotypes, a certain degree of phenotypic variability has been observed, which was the most striking for 35delG homozygous patients. The severity of hearing loss in 35delG homozygotes showed significant inter- and intrafamilial phenotypic variability, ranging from mild or moderate to severe and profound [61]. In a recent study, involving a set of 1277 35delG homozygous patients from 14 countries, Hilgert and colleagues [64] sought to identify modifier genes for connexin 26-related hearing impairment. A whole-genome association (WGA) study has been performed on 35delG homozygotes by comparing the genotypes of mild/moderate cases and profound cases. The data suggest that the phenotypic variability in 35delG homozygous patients cannot be explained by the effect of one major modifier gene and more likely caused by a smaller effect of different interacting genes.

### Dominant Modifier DFNM1, A Suppressor of the Profound Hearing Loss Locus DFNB26

Homozygosity mapping within consanguineous families is a powerful method for identifying genes that modify deafness phenotypes. In a consanguineous Pakistani family, Riazuddin and colleagues [3] mapped an autosomal recessive nonsyndromic hearing loss locus, *DFNB26*, to chromosome 4q31. Homozygosity for the *DFNB26*-linked markers can lead to profound congenital deafness. However, there were 7 unaffected family members who were also homozygous for the *DFNB26*-linked haplotype and thus were nonpenetrant. In this rare instance, the family size was sufficiently large to allow the investigators to map *DFNM1*, a single dominant suppressive locus of *DFNB26*, to chromosome 1q24 (near D1S2850). Identification and functional analyses of these 2 genes should reveal the molecular pathophysiology of *DFNB26* and elucidate the underlying mechanism of *DFNM1* suppression. Of note, the map location of *DFNM1 *is within the 22-cMorgan interval that contains the *DFNA7* locus [65], suggesting that the *DFNM1 *suppressor phenotype and *DFNA7 *deafness may be two phenotypic variants of the same gene. Also, located within the *DFNM1 *interval, the *PMX1 *(paired mesoderm homeobox) gene that is expressed in the cochlea, is a potential candidate gene [66].

### The *ATP2B2* Gene, A Modifier of *CDH23* Deafness

*CDH23* encodes cadherin 23, a putative calcium-dependent adhesion molecule required for proper morphogenesis of cilia of the hair cells of the inner ear. Mutations in the Cadherin 23 gene (*CDH23*) have been associated with Usher syndrome type 1D, and DFNB12, a form of autosomal recessive hearing loss [67]. In homozygous waltzer mice, mutations in *Cdh23* cause profound deafness and vestibular dysfunction [68]. Interestingly, subsequent evidence shows that variants in *Cdh23* also underlie the age related hearing loss (*Ahl)* locus and contribute to differences in onset and severity of AHL among inbred mouse strains [69]. Thus, Cdh23 may be an important gene that may not only underlie certain forms of congenital deafness but also age-related hearing loss and has the potential to genetically interact with other deafness genes to affect hearing. The finding that variants of the *Atp2b2* gene, which encodes a calcium pump in the plasma membrane, module the severity of hearing loss in waltzer mice [70] would add further support to the contention. A nucleotide change in the human *ATP2B2* gene was later shown to exacerbate hearing loss in individuals homozygous for a *CDH23 *mutation, similar to the Atp2b^*2dfw-2J*^–Cdh23^*753A/G *^interaction affecting hearing in mice, in a consanguineous family of the European descent [71]. In this family, five adult siblings expressed variable degrees of hearing loss differentially affecting the higher frequencies. While all individuals with hearing loss were homozygous for a missense mutation (F1888S) in *CDH23*, those subjects with the hearing loss affecting all frequencies were also heterozygous for a missense mutation (V586M) in plasma-membrane calcium pump PMCA2, which is encoded by *ATP2B2*. Individuals with hearing loss in the higher frequencies but with normal thresholds in the lower range had the normal 586V allele. Thus, the 586M allele of *ATP2B2 *seems to have a clear modifying effect when combined with the *CDH23* mutation. Interestingly, heterozygosity for the *ATP2B2* variant was not sufficient to cause hearing loss — an observation that is typical of most modifier genes: they generally have detectable effects on the trait, only in the presence of their target gene variants.

## MODIFIERS OF HEARING LOSS IN MICE

Because of their diverse genetic background, inbred strains of mice manifest strikingly different patterns of background pathology. A specific phenotype can even vary in one mouse strain or disappear in another strain. To detect a modifier it is required to cross the mutant allele with different inbred strains to permit random segregation of the mutant and modifying alleles. Analyses of appropriate linkage crosses and congenic lines can be used for the mapping of modifier loci, and the major modifying loci can be identified by positional-candidate gene method. In the following section, we report an evidence for modifiers of hearing impairment in mice. 

### Modifiers of Deaf Waddler (*mdfw*)

Mice homozygous for the deaf waddler mutation (dfw/dfw), localized on chromosome 6, exhibit circling behavior as a result of vestibular dysfunction in addition to congenital hearing loss. A plasma membrane ATPase type 2-Ca^2+^ transporter pump (*Atp2b2*) has been identified as the gene responsible for the *dfw* mutation. Several spontaneous mouse mutants of *Atp2b2* have been reported, including the deafwaddler (Atp2b2^dfw^), deafwaddler-2J (Atp2b2^dfw2J^), wriggle mouse Sagami (Atp2b2^wri^), and tommy (Atp2b2^tmy^). The *dfw* mutant results from a single A->G nucleotide transition leading to a glycine to serine substitution at residue 283 of PMCA2; in dfw-2J, a 2 bp deletion causes a frameshift and premature stop codon after residue 471 of PMCA2; in wri, a G->A transition replaces a glutamate by a lysine in a highly conserved region in the fourth transmembrane domain of PMCA2; and in tmy, another G->A transition generates a change from glutamate to lysine in the ATP binding domain of PMCA2. The vestibular abnormalities in *dfw* mice are less severe than in *dfw-2J*, *wri*, or *Atp2b2*-null mice, which express no functional PMCA2. The *dfw* mutation results in a reduction in Ca^2+^ transport activity to 30% compared to that of the wild-type activity of PMCA2 [72]. The localization of the ATP2B2 protein to stereocilia and basolateral wall of hair cells, suggests that it provides the means to remove calcium from both auditory and vestibular hair cells [73]. The onset and severity of hearing loss in heterozygote *Atp2b2*^+/-^ vary with the genetic background. This variation is attributable to a locus named modifier of deaf waddler (*mdfw*), which maps to mouse chromosome 10, near two other loci affecting hearing in mice: the ahl locus and the mouse mutations waltzer (*v*). There are two known mutant alleles of *mdfw*: the CAST/Ei – derived *mdfw* allele protects *dfw*/+ mice from deafness in a dominant fashion, whereas the recessive cBy-derived *mdfw* allele permits hearing loss to occur in *dfw*/+ mice. A fine structure map analysis has subsequently suggested allelism between *mdfw* and *Cdh23^v^*[74] and a single nucleotide polymorphism in *Cdh23*, Cdh23^753A/G^ associated with *Ahl1* was identified as the molecular basis of *mdfw* [70]. The hypomorphic *Cdh23*^753A^ allele causes skipping of exon 7 leading to an in-frame deletion of 43 amino acids at the extracellular amino-terminus. The *Cdh23*^753A^ variant is generally associated with increased susceptibility to AHL but the predisposition conferred by *Cdh23^753A^* depends on the effects of several strain-specific genetic factors, including the mitochondrial mutation *mt-Tr*^9827ins8^ and the *ahl2* and *ahl3 *loci [70]. The *Cdh23* locus has also been shown to modify the deafness caused by the *Mass1^frings^* mutation in BUB/BnJ strain mice and can account for the phenotypic manifestations differences observed between Frings (*Cdh23^753G^*) and BUB/BnJ (*Cdh23^753A^*) [75,76].

### Modifiers of Deafness in the *tub *Mouse

A recessive mutation in the *tub* gene causes obesity, early-onset cochlear and retinal degeneration in tubby mice [77]. The gene encodes a member of the Tubby family of bipartite transcription factors [78]. The phenotypes observed in tubby mice are affected by the genetic background [77], but the exact mechanisms by which the *tub *allele causes the tubby phenotypes is unknown. A major dominant modifier locus of tubby hearing 1 (*moth1*) has been identified on chromosome 2 by linkage analysis [77]. On the coisogenic C57BL/6J background, homozygous tubby mice suffered from profound deafness. When crossed onto the AKR/J and CAST/Ei backgrounds, *tub/tub* exhibit a wide spectrum of hearing thresholds ranging from deafness to a complete rescue. Amino acid polymorphisms in the microtubule-associated protein 1A gene (*Mtap1a*) have subsequently been found responsible for the modifying effect of *moth1* [79]. The *Moth1* modifier influences only the hearing phenotype of tubby mice and has no effect on any other phenotypic manifestations of the tubby mutation, such as retinal degeneration and obesity. As MTAP1A may be involved in trafficking of synaptic components from the cytosol to the synaptic junction [80,81], it was suggested that the function of Tub may be associated with synaptic function between hair cells and cochlear neurons [79]. 

## DIGENIC INHERITANCE - A CAUSE OF SOME FORMS OF DEAFNESS

Most cases of genetic deafness result from mutations at a single gene, but an increasing number of examples are being recognized in which recessive mutations at two loci are involved. In digenic inheritance, mutations in each of two unlinked genes are present in a single individual, and the combination of the two genetic hits causes a disease phenotype that is not apparent when an individual carries only one of these gene alterations. This mechanism complicates genetic evaluation and counseling, but provides an explanation for individuals with a heterozygote mutation in a deafness gene who, for previously unknown reasons, are deaf. Because, difference between modifier genes and diginic inheritance is not always clear, we have included in this review several examples of digenic inheritance of deafness that have been reported in both humans and mice. Given the high degree of similarity between mouse and human genomes, it came as no great surprise that some forms of digenic inheritance and genetic modifications are found in both species. 

Digenic interactions are known to be an important cause of deafness in patients who carry a single mutation at the gap junction protein *GJB2* (*connexin 26*) along with a deletion involving the functionally related *GJB6* (connexin 30). The deafness of individuals heterozygous for *GJB2* mutations has been associated with in trans heterozygosity for *GJB6 *gene deletions [82,83]. Double heterozygous mice for both *Gjb2* and *Gjb6* mutations (Gjb2^+/−^ Gjb6^+/−^ double heterozygotes) exhibit a moderate hearing impairment and reduced endocochlear potential compared with single heterozygotes [84], in contrast with the phenotype observed in humans, wherein most double heterozygotes for del(GJB6-D13S1830) and a *GJB2* mutation have severe or profound hearing impairment [82,83,85-89]. Interestingly, the del(*GJB6-D13S1830*) allele is the most frequent in Spain, France, the United Kingdom, Israel, and Brazil, accounting for 5.0–9.7% of all the *DFNB1 *alleles [62,90] and this finding provided an explanation for the deafness in as many as 30% to 70% of affected *GJB2* heterozygotes in those countries. However, this large deletion has not been detected in Turkish, Italian, Austrian, Greek Cypriot and Chinese nonsyndromic hearing loss patients [90-94], suggesting that other mutations, both within DFNB1 and elsewhere may be involved in epistatic interactions with *GJB2*. We recently provided an evidence that mutations in the *Cx26* and *Cx31 *genes can interact to cause hearing loss in digenic heterozygotes. Direct physical interaction of the two proteins is supported by data showing that Cx26 and Cx31 have overlapping expression patterns in the cochlea and that *in vitro* the two connexins were able to co-assemble in the same junction plaque [15].

Recessive mutations in genes coding for cadherin 23 (*Cdh23*) and protocadherin 15 (*Pcdh15*) cause deafness in both mice and humans. Given the similarities in function and effect of pathogenic mutations of *Cdh23* and *Pcdh15*, we assessed whether there is functional interaction between the two genes by generating double mutant mice for *Cdh23 *and *Pcdh15* (14). Our data showed that mice doubly heterozygous for *Cdh23^v-2J^* and *Pcdh15^av-3J^* mutations exhibit age related deafness and abnormal stereocilia in outer and inner hair cells of the organ of Corti, whereas single heterozygotes lack this pathology. We also had evidence of a digenic inheritance of mutations in *CDH23* and *PCDH15* in three unrelated families with USH1 phenotype. Other additional cases of digenic inheritance of deafness have been reported in humans including an interaction between the *GJB2* and the mtDNA 12S rRNA (*MTRNR1*) gene, in which the hearing loss associated with the 1555A>G mitochondrial mutation is more severe in patients who also are heterozygous for the *GJB2 *mutation [49]. Interaction between mutant alleles at the DFNA12 and DFNA2 loci was proposed to explain the phenotype variation in an extended Swedish family with nonsyndromic progressive bilateral sensorineural hearing loss. Analysis of the phenotypes and haplotypes shared by the affected individuals indicate that the two loci are responsible for the hearing impairment in the family and the presence of both of them is associated with a more severe hearing loss as compared to the hearing loss caused by each of the loci separately [95]. In a family of Jewish Yemenite origin, a mutation in *MYO7A *appears to exacerbate deafness in individuals with two mutant USH3 alleles, suggesting an interaction between the gene products [96]. USH3 encodes clarin-1, an integral membrane protein expressed in cochlear hair cells and ganglion cells, suggesting that the USH3A–MYO7A interaction may play a role in hair cell synapses [97].

## STRATEGIES FOR IDENTIFICATION OF MODIFIER GENES

Several approaches can be adopted for mapping and identifying the modifier genes for human disorders. The family-based linkage strategy can be applied to investigate the phenotypic difference among affected individuals from multiplex families with significant intrafamilial variation. Using this method, the *DFNM1* locus, a modifier of *DFNB26-*linked deafness was identified [3] and the deafness phenotype associated with the 1555A>G mutation in *MTRNR1* was shown to be modified by three different genes: *MTO1*, *TFB1M* and *GTPBP3* [51,52]. Another approach is to perform disease-marker association studies in unrelated patients. The ability to genotype hundreds of thousands of single-nucleotide polymorphisms (SNPs) covering the complete genome makes the whole-genome association (WGA) scans feasible due to innovative combinations of assays and array platforms. However, WGA studies are costly, if samples are individually genotypes. A cost effective solution is to genotype pooled DNA on SNP microarrays and screen for allelic frequency differences between cases and controls (or low versus high groups). This approach has been used successfully to identify SNPs associated with complex disorders [98-102] and recently applied to identify modifier genes for Connexin 26-related hearing impairment in a set of 1277 35delG homozygous patients from 14 countries. The study suggests that the phenotypic variability in 35delG homozygous patients cannot be explained by the effect of one major modifier gene [64]. A limitation of pooling-based WGA studies is that they are only effective in identifying common variations with a large effect on the disease.

In mice, the most efficient strategy for mapping modifier genes is to cross parental inbred strains that carry the causative mutation and show a difference in phenotype. To ascertain potentially dominant modifier effects, homozygous mutants are generally used to generate the F1 hybrids, which leads to a genetically uniform population. Two genotypes in F1 hybrids are obtained, which provides information of effects of genetic background that are not due to the mutant allele, if a cross is initiated with heterozygous mutants. An intercross of F1 hybrid carriers produces a higher degree of allele combinations and increases the chance to detect phenotypic differences and epistatic interactions. For dominance effects, in which a modifier affects the mutant allele only when in a homozygous state, the backcross is more suitable as it yields a larger phenotypic population for linkage detection. The intercrosses (F2 animals) and backcrosses (N2 animals) progeny are assessed for phenotypic variation. Standard quantitative trait loci (QTL) analysis methods can then be applied to map the modifier loci in F2 animals with modified phenotypes [103]. The use of animals with extreme phenotypes for initial linkage analysis will maximize information without affecting detection sensibility [104]. If a major modifying locus is detected, a combination of conventional fine mapping in a large F2 intercross and progeny testing can be used to narrow the genetic interval to provide a basis for positional cloning [77,79]. When the fraction of the phenotypic variance is due to multiple loci, production of congenic lines may be needed to identify the modifier loci. Crosses for fine-resolution mapping may subsequently perform on congenic line harboring a phenotypic effect of the modifier locus greater than the non-genetic variation. Conventional positional cloning strategy can then be applied, once a high-resolution map has been obtained [103,105]. Current technology, such as gene expression microarray analysis may be combined with the use of congenic lines to directly identify a mutant modifier allele or point to the misregulation of a pathway in which the modifier gene plays a role [106]. 

## CONCLUSION

The past decade has witnessed great advancements in our knowledge of the structure and function of genes involved in deafness. However, many of these genes do not follow the rules of the typical Mendelian expression patterns of inheritance and may be subject to the effects of modifier genes. In this review, we have presented the examples of modifier loci and/or their genes for hereditary hearing loss that have been discovered in both humans and mice. While chromosomal location of some modifier effects has been identified, cloning of modifier  genes still remains to be a difficult task. The challenge of molecular mapping and identification of modifier loci will propel development of newer strategies. The elucidation of modifier genes will provide insight into molecular and cellular pathways and mechanisms of genetic interactions involved in auditory systems. Furthermore, it may also be helpful in designing new therapeutics that target expression of modifiers.

## ABBREVIATIONS

ADNSHL: = Autosomal dominant non-syndromic hearing loss
ARNSHL: = Autosomal recessive non-syndromic hearing loss
CX26: = Connexin 26
DFNB: = Autosomal deafness nonsyndromic recessive locus
DFNM: = Deafness nonsyndromic modifier
GTPBP3: = GTP binding protein 3 (mitochondrial)
HL: = Hearing loss
K^+^: = Potassium
MTO1: = Mitochondrial translation optimization 1 homolog (S. cerevisiae)
mtDNA: = Mitochondrial DNA
NSHL: = Nonsyndromic hearing loss
QTL: = Quantitative trait loci
ROS: = Reactive oxygen species
rRNA: = Ribosomal RNA
SNPs: = Single-nucleotide polymorphisms
TFB1M: = Transcription factor B1, mitochondrial
tRNASer: = Transfer RNA for serine
tRNAGlu: = Transfer RNA for glutamic acid
tRNAArg: = Transfer RNA for arginine
tRNAThr: = Transfer RNA for threonine
TRMU: = tRNA 5-methylaminomethyl-2-thiouridylate methyltransferase
WGA: = Whole-genome association
